# The evolution, impact and properties of exonic splice enhancers

**DOI:** 10.1186/gb-2013-14-12-r143

**Published:** 2013-12-20

**Authors:** Eva Fernández Cáceres, Laurence D Hurst

**Affiliations:** 1 Department of Biology and Biochemistry, University of Bath, Bath BA2 7AY, UK

## Abstract

**Background:**

In humans, much of the information specifying splice sites is not at the splice site. Exonic splice enhancers are one of the principle non-splice site motifs. Four high-throughput studies have provided a compendium of motifs that function as exonic splice enhancers, but only one, RESCUE-ESE, has been generally employed to examine the properties of enhancers. Here we consider these four datasets to ask whether there is any consensus on the properties and impacts of exonic splice enhancers.

**Results:**

While only about 1% of all the identified hexamer motifs are common to all analyses we can define reasonably sized sets that are found in most datasets. These consensus intersection datasets we presume reflect the true properties of exonic splice enhancers. Given prior evidence for the properties of enhancers and splice-associated mutations, we ask for all datasets whether the exonic splice enhancers considered are purine enriched; enriched near exon boundaries; able to predict trends in relative codon usage; slow evolving at synonymous sites; rare in SNPs; associated with weak splice sites; and enriched near longer introns. While the intersect datasets match expectations, only one original dataset, RESCUE-ESE, does. Unexpectedly, a fully experimental dataset identifies motifs that commonly behave opposite to the consensus, for example, being enriched in exon cores where splice-associated mutations are rare.

**Conclusions:**

Prior analyses that used the RESCUE-ESE set of hexamers captured the properties of consensus exonic splice enhancers. We estimate that at least 4% of synonymous mutations are deleterious owing to an effect on enhancer functioning.

## Background

The identification of splice sites in long metazoan transcripts requires not just splice site sequences. Indeed, it is estimated that only around 50% of the information specifying splice sites is at the splice site itself [1]. In addition there are short stretches within the immature RNA that function as either enhancers or suppressors of splicing. These can be either within the exons or the introns. Here we concentrate on the exonic splice enhancers (ESEs). ESE motifs function in part by binding SR proteins to aid exonic splice site recognition [2,3]. In addition they may function to help retain unspliced pre-mRNAs in the nucleus [4].

We concentrate on ESEs because, unlike exonic splice suppressors [5], ESEs are claimed to have a profound influence on protein and gene evolution [3,5-7]. ESEs are thought to be enriched near splice sites [8-10], potentially explaining why exon ends are slower evolving at both synonymous [6] and non-synonymous sites [5] and why SNP density is lower [10,11]. Closer analysis indeed supports the view that this slower evolution is in large part owing to the impact of purifying selection on ESEs in proximity to exon ends [5-7,10,11]. Consistent with this, SNPs responsible for altered splicing are enriched at exonic ends [10]. ESEs appear to be under particularly strong selective constraint up to 50 to 100 bp from exon ends [11]. As the average human exon is approximately 130 bp, this means that for many human exons all of the exon is ‘exon-end’ in the sense that it is a domain in which ESEs can be functional. Given the possibility that ESEs are at a high density (approximately 30% to 40%) at exon ends and that they are evolutionarily conserved, their impact on amino acid and codon usage is of considerable interest to molecular evolutionists.

However, most of the above work comes with a potentially serious caveat, in that nearly all prior work on the evolutionary impact of ESEs has employed the same set of putative ESEs (for an exception see [10]), that deduced by Fairbrother et al. [9,12]. Given that there now exist three other systematic attempts to define sets of putative ESEs, it is relevant to ask whether these sets agree on the properties of ESEs and to what extent they concur as to which hexamers function as ESEs. Considering the sets of hexamers agreed on, at least in part, by the various methods also provides an opportunity to characterise the properties and impact of ESEs. Such issues are not only of interest to the molecular evolutionary community. If, for example, ESEs are under purifying selection then mutations disrupting ESEs are possible causes of splice-associated diseases [3]. Understanding the commonality of purifying selection on ESEs is then of relevance to medical genetics and diagnostics.

There have been several different approaches to describe sequences that function as ESEs. Early in-depth approaches identified ESEs by looking for splice-altered disease alleles [13], by mutagenesis of mini-genes and by systematic evolution of ligands by exponential enrichment (SELEX) [14-16]. Given the binding motifs of a series of SR proteins, applications such as ESEfinder attempt to identify possible ESEs in any given sample of sequence [17]. We do not consider these analyses but rather concentrate on the four systematic attempts to define ESEs.

The majority of systematic attempts to define ESEs employ computational approaches, confirmed with experimental support. Typically these approaches start with a presumption about the distribution of ESEs and look for the sequences most enriched in these trends. For example, Fairbrother et al. presumed that ESEs will be enriched in constitutive exons compared with introns and more abundant in exons with weak splice sites than in those with strong splice sites [9,12]. Looking for 6-mers enriched on both of these axes led to a candidate set of ESEs. A similar approach, but one avoiding potential confounding with amino acid coding, was taken by Zhang and Chasin [18]. This group identified motifs enriched in internal non-coding exons of protein coding genes compared to unspliced pseudo-exons and 5’ untranslated regions. Goren et al. [19] took an alternative approach and, supposing that functional ESEs should be slow evolving, looked for motifs that were more conserved than expected at synonymous sites. Combining this with evidence for enrichment compared with background codon usage rates led to the identification of a set of exonic splice regulatory motifs, the majority of which proved on experimental confirmation to be ESEs. While a minority were exonic splice inhibitors, the precise numbers are uncertain not least because ESEs can also function as exonic splice inhibitors depending on their position and context within the exon [20].

While these three predominantly computational approaches have provided an extensive compendium of ESE sequences it is possible that they are not exhaustive. Given, too, the possibility of conditional and position dependent effects, recently Ke et al. [20] adopted an experimental high-throughput approach by considering the effects of all possible 4,096 6-mers at five locations in two model exons. Taking into account overlap sequences this permitted the identification of numerous ESE hexamers.

Here we consider the ESEs provided by the four analyses so as to ask whether there is now any general consensus on the properties of ESEs and to ask whether any dataset may be particularly different from the others. We start by considering the overlap between the four groups. Then we consider a series of possible diagnostics of ESEs.

First, we ask about purine content. The earliest experimentally determined ESEs tended to purine rich [9,21-23]. Indeed, the binding sites of the SR protein SF2/ASF, for example, are over 80% purine [24]. The pure purine hexamer GAAGAA appears to be one of the strongest ESEs [9]. Subsequently some AC-rich motifs and pyrimidine motifs have, however, been identified [14,25].

Second, we ask about intra-exon location. A considerable body of work implicates ESEs as functioning close to exon junctions [8-10], not least the finding that SNPs demonstrated to alter splicing, but not at splice sites, tend to be close to exon boundaries [10]. We thus ask about the trend in usage of ESEs in the vicinity of junctions. Specifically, we ask whether their usage declines as one moves towards the core of the exon. Similarly, it has been noted that the relative usage of many synonymous codons changes as one approaches exon-intron junctions [26,27]. Usage of a codon within an ESE (as defined by RESCUE-ESE) predicts these trends [26,27]. We thus employ a previously derived statistical method, HPI [5], to establish the usage of each codon within any given set of ESEs and then ask whether the set of ESEs is then consistent with observed trends in relative usage of pairs of synonymous codons.

We additionally ask whether ESEs impose purifying selection at synonymous sites. If the ESE motifs are functional they should probably be under purifying selection most of the time, this presumption underpinning the method of Goren et al. [19]. Analysis of the RESCUE-ESE motifs supports this position [5-7,10,11]. To address this we ask about the rate of evolution of four-fold degenerate synonymous sites in and out of ESEs as a function of the distance from the exon ends, controlling for differential nucleotide usage. In addition, and related, we ask about the frequency of SNPs in ESE and non-ESE sequence, under the expectation that ESEs should harbour fewer SNPs [7,11], not because they mutate less, but rather because purifying selection should remove them from a population or force them to low frequency (making them less likely to be evidenced).

An association between ESEs and with weak splice sites is commonly assumed (for example, [9]). This is expected, as it is thought that ESEs can be under selection to compensate for weak splice sites [28], a feature that in turn is dependent in part on the length of the flanking intron [29]. We thus consider whether ESE density is higher in vicinity to weak splice sites and longer introns.

We find strong evidence that ESEs impose purifying selection on exonic ends. Given this and given that conserved alternative exons tend to be slow evolving [6,30,31], we ask whether this might reflect a higher density of ESEs in either alternative exons or in conserved exons. Finally, we use information gathered to estimate the proportion of synonymous mutations that are under selection owing to their disrupting ESE function. We start by outlining the four methods in more detail so as to clarify the possible biases intrinsic in each approach.

## Methods

### The datasets

#### ***RESCUE-ESE***

This dataset was obtained using a computational approach to look for hexamers with ESE activity [9,12]. The authors make two assumptions: (1) ESEs should be enriched in constitutively splicing exons and avoided in flanking intronic sequences; and (2) ESEs must have a higher frequency in exons with a weak splice site than in exons with a strong one.

To compute this dataset the authors employed 4,817 human genes containing 31,463 introns and 28,933 internal exons. Exons smaller than 200 bases long were considered in their entirety. Of those introns and exons longer than 200 bases only the 100 first and 100 last bases were considered. The results were experimentally validated. External comparisons using prior data were also employed (for example, from published mutationally characterised natural ESEs and binding SELEX analyses). Although the predictions offered by RESCUE sets were evaluated as valid using different methods, the authors argue that some sequences with ESE activity were not reported by RESCUE method.

This ESE motif set contains 238 hexameric candidates, of which 103 were described as ESE at 5’ exon ends and 198 at 3’ ends, with 63 motifs common to both sets. This complete hexamer set is 6% out of 4,096 possible hexamers. They were clustered based on sequence similarity providing 10 different consensus candidates.

It is not immediately obvious that the protocol employed by Fairbrother et al. initiates any bias as regards our tests, baring enrichment in exons *versus* introns and association with weak splice sites, these aspects being definitional. Note that while these ESEs are defined as being enriched in the last 100 bp compared with introns, it does not follow that they need be enriched close to splice sites when compared with other parts of exons. Thus it is not obvious that this method necessarily predicts that ESEs will decline in usage as one moves away from exon junctions, the test that we perform. While the team required enrichment in constitutive exons as a definitional property, they also rely on enrichment near weak splice sites, which may be more common in alternative exons. The method may thus be biased either towards discovering motifs associated with alternative exons or with constitutive exons.

One possible confounder is a difference in nucleotide content owing to the definition requiring enrichment in exons compared with introns, especially given the polypyrimidine track often seen in introns necessary for splicing. However, although C is rare in RESCUE ESEs it is actually more common in the terminal 100 bp of exons than in the terminal 100 bp of introns (23% *versus* 20%), suggesting that the protocol has not led to a *de facto* enrichment for pyrimidines or purines. There is nothing intrinsic in the method to predict sequences that, controlling for nucleotide content, are slow evolving. There is no obvious reason why this method would predict relative synonymous codon usage in the vicinity of splice junctions any differently from any other method that looks for enrichment in exons compared with introns.

#### ***PESE***

This method focused on describing ESE motifs avoiding biases due to protein coding sequences [18]. Since there are differences in sequence composition between exons and introns, the authors dealt with that problem by just considering non-protein-coding exons. They compared frequencies of octamers over-represented in constitutively spliced non-coding exons *versus* unspliced pseudoexons and 5’ untranslated regions (UTRs) of intronless genes. The authors assumed that ESE motifs are not frequent in pseudoexons and that UTRs lack ESE activity.

In this dataset 502 non-coding exons that have <70% inclusion, 2,309 pseudo-exons adjacent to the non-coding exons and 864 5’ UTRs of intronless genes were used. Due to the low number of sequences remaining after filtering, comparisons were performed allowing one mismatch (out of 8 nucleotides).

This dataset gathers 2,069 putative ESE motifs out of 65,536 possible octamers (around 3.2%). These motifs were clustered in 80 PESE families according to sequence similarity. The team then deduced a set of 238 hexamers taking just those motifs that appeared at least seven times.

The authors recognised one evident bias in their dataset [18]. As UTRs are rich in CpGs, by requiring their motifs to be enriched compared with these UTRs, by necessity they biased towards CpG poor ESEs. In some ESEs that they compare their set to CpG content is around 10% but is 4.2% in their set, rather closer to the exonic mean of 2.8%. This bias may impact estimates of rates of evolution (if uncontrolled for nucleotide content) and synonymous codon usage trends. The requirement for octamers to be over-represented in constitutive exons will provide a bias. Experimental confirmation of the role of many of the ESEs was subsequently provided [32].

#### ***ESR***

This dataset is composed of 285 hexameric exonic regulatory sequences, both ESE and ESS motifs, using a computational approach [19]. The authors selected those sequences that: (1) were more frequent than the random expectation, taking into account the relative codon frequency; and (2) were highly conserved between human and mouse at the wobble positions. Only human-mouse orthologous exons with the same length, shorter than 250 nucleotides and with classical GT-AG splice sites were considered, leaving 46,103 exons.

This set is, by definition, biased towards finding slow evolving sequence. One of our tests, that based on the rate of synonymous site evolution, is thus void. As C tends to be the mutable residue this set is likely biased against C.

#### ***Ke-ESE***

Ke et al. provide what, at first sight, should be the gold standard for ESEs, it being the first systematic fully experimental analysis [20]. The authors [20] carried out an assay where all possible hexamers (4,096) were substituted at five positions within two different internal exons in a mini-gene construct. Constructs were transfected into human cells and the transcripts sequenced thereby detecting correct splicing. The splicing activity in every hexamer was quantified taking into account the different positions and the context effect.

This datasets contains 1,182 motifs reported to have splice promoting activity, that is, 29% of all possible hexameric combinations. Every sequence was assigned a score depending on the strength of splice activity. Many functional hexamers have a low score. To provide a better representation of the strong hexamers the team also considered the top 400. This dataset we refer to as Ke-ESE400. It is possible that this experimental set is biased to finding hexamers that function in proximity to weak splice sites, as the exonic ends were modified to decrease the rate of splicing, thus permitting assessment of the impact of hexamers in close proximity to the splice sites. Although we cannot find details of the dimensions of the construct, the introns appear small (see their Figure one A). This too might provide a bias as the process of splicing for short and long introns may be qualitatively different [33].

### Obtaining ESE datasets

We downloaded the ESR motifs (their Table S2) and Ke-ESE motifs (their supplementary Table 4) from the original papers. For Ke-ESE_top400 we selected the top 400 hexamers with the highest scores. RESCUE-ESE dataset was downloaded from [34] and PESE original octamers from: [35]. PESE hexamers were extracted from octamers with a minimum of seven occurrences.

### Determining the extent of intersection

To define the intersection between any two sets of ESE motifs, we determine how many of the 6-mers are common to both sets. If, for example, the 6-mer GGTACG is reported to be present in both focal datasets, then it is considered to be part of the overlap/intersection set. Note we do not consider ‘overlap’ here to mean that the motifs are overlapping, but rather that the identical motif is present in the two sets. For example, while AGGTAC overlaps with the motif GGTACG, in the sense that we can perfectly align five of the base pairs, the two motifs are not identical. This would not count as evidence of overlap/intersection between the two datasets.

### Assessment of enrichment near exon boundaries

To assess the location of hexamers we follow the method of Zhang and Chasin [18]. We take each exon larger than 50 nt and create a new merged sequence which contains: 100 nt from flanking intron 5’, 100 nt from exons, and 100 nt from flanking intron 3’. Taking into account that exons have different lengths, we just take 50nt from each end and 100 nt from the middle of the exon if the exon was longer than 200 nt. These merged sequences were used to calculate the frequency of ESE motifs per position for each dataset. We also tested whether there are significant differences between the centres of exons and the ends.

### Human-mouse orthologous internal exons

We downloaded the human-mouse orthologous genes from Ensembl (157,061 files) keeping just those assigned as ortholog_one2one (121,592 files). We eliminated duplicated genes keeping the longest human transcript. In those instances where the human transcripts were of equal size we retained the one with the longest mouse transcript. These filters resolve the dataset down to 15,948 files. For each CDS, we removed all those which did not start with ATG, which did not end with a stop codon, which were not a multiple of 3, that had any internal stop codons or that had non-standard bases. We also removed those with a different number of exons in human and mouse or whose orthologous exons differed more than 5% over the exon length. In addition we excluded from analysis the first and last exons. Where relevant, we trimmed exons so that they started and ended in frame.

### Alignments of orthologous exons

The alignment was performed at amino acid level using MUSCLE [36]. Afterwards, the peptide alignment was used as a template to reconstruct the nucleotide alignment from the original nucleotides.

### SNP analysis

SNPs were extracted from dbSNP build 137 [37] using biomart. The number of exons analysed was 17,170. For each position away from an exon junction, we first considered whether it belonged to an ESE or not, given the dataset in question. Then for the ESE and non-ESE sets we considered the proportion of sites across exons that contained one or more SNPs. If a position within a given exon has more than one SNP, just one is considered (that is, we are interested in the proportion of sites with one or more SNPs, not the number of SNPs nor their frequency).

### HPI calculation

To assess the ability of each ESE dataset to predict relative synonymous codon usage, for each codon we calculated a Z score, termed the hexamer preference index (HPI), as detailed previously [5]. To understand the extent to which any given codon is found in a given ESE set, it is necessary to recognise that the ESEs can appear in any frame. Considering then a series of six nucleotides, n1n2n3n4n5n6, codons n1n2n3, n2n3n4, n3n4n5 and n4n5n6 are specified completely. We sum all such complete codons for all splice enhancer hexamers within any given putative ESE set. This is equivalent to assuming that each hexamer appears in each frame at equal rates. This provides a metric of ESE hexameric involvement of all possible codons, within any given ESE dataset. The three stop codons were removed and the proportions re-normalised. The usage of each codon within the list of ESEs was then compared to that expected given the codon usage within the genome. To do this, we normalised (after stop codon removal) the relative abundances of all codons as specified in the human codon usage database [38]. We then generated 10,000 sets of random hexamers, each set being the same size as the focal ESE list. Random hexamers were generated by joining two codons selected at random in proportion to their frequency in the genome. We parsed each random hexamer in the same manner as we parsed the input list, extracting all non-stop codons.

For each codon we determined the mean and standard deviation in relative abundance across the random sets. The difference between the observed frequency of a codon in the real hexamer set and in the randomised sets, normalised by the standard deviation across the randomised sets, is then the Z score, termed HPI. A high Z score implies a codon particularly enriched, compared to its usage in the genome, in the relevant ESE dataset.

### Rate of gene expression

The mean expression of 11,449 genes in 28 human tissues was derived from BioGPS, this corresponding to the data from the Affimetrix array analysed by Su et al. [39]. We summarised GCRMA normalised probe intensity levels to Ensembl IDs corresponding to protein coding genes. Probes matching more than one gene ID were discarded. We applied a mask to all expression values lower than the average of the expression of the negative controls in each tissue, transforming them to 0. Genes with expression values lower than the average of the negative controls in every tissue were removed. Expression values where then normalised against the total signal level in each tissue. We consider expression breath (the proportion of tissues within which a gene is expressed), tau (a measure of skew in expression), mean expression level and median level.

## Results

### The degree of concordance between datasets

If all datasets were describing the same kind of motifs we would expect to find a high proportion of ESE motifs that appear in all sets. Some discordancy is nonetheless expected by the fact that the methods used to identify the motifs are different and from the fact that all methods may be subsampling from the same larger set of true ESEs.

**Figure 1:**
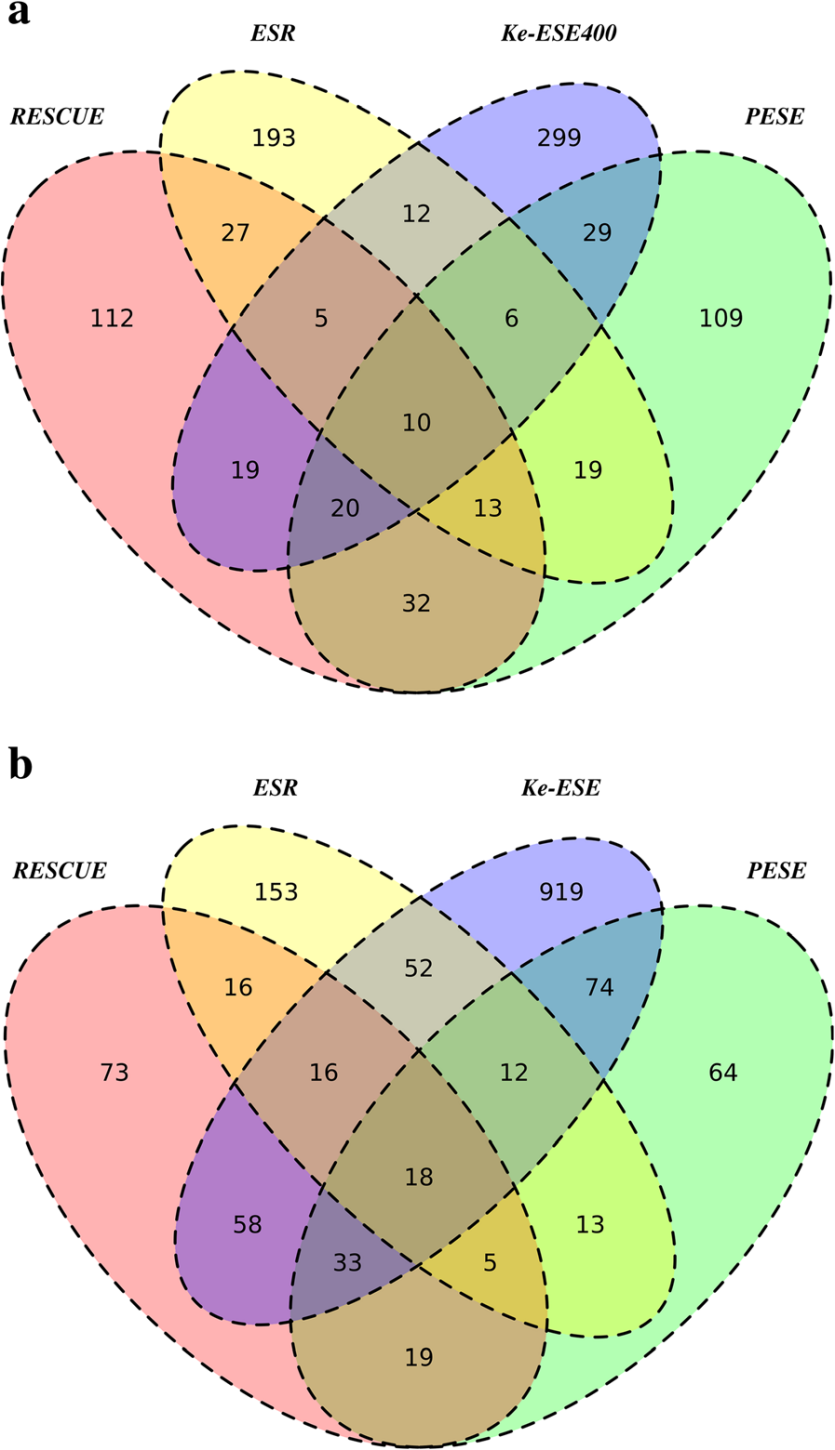
**Venn diagram showing overlap between datasets in hexamers identified as possible ESEs (a) using Ke-ESE400 and (b) Using Ke-ESE.** The great majority of ESEs are unique to any given dataset. For example, 47.06%, 45.80%, 67.72% and 74.75% are motifs unique to for RESCUE, PESE, ESR and Ke-ESE400, respectively.

From simulation we expect, under the assumption that the four datasets are independently drawn from the set of 4,096 hexamers, an overlap of about 1 ESEs between the four sets (where Ke-ESE is represented by Ke-ESE400). We find significantly more than this, with 10 of 905 nominated hexamers (1.1%) shared by all four datasets (Figure 1). While this is greater than the null (*P* <0.001) it appears modest. Indeed, if we consider the set of 905 different motifs nominated as possible ESEs and randomly sample from this pool to generate four random datasets, the extent of overlap is no greater than expected by chance (*P* = 0.25), the expected degree of sharing under this random null being 0.97% (that is, between 8 and 9). We can also ask the inverse question. We can suppose there are only 400 true ESE motifs and that Ke-ESE400 has found them all. If the remaining three datasets are samplings of these 400, we expect about 100 motifs to be in common between all four, not just 10. However, this result, while very highly significant, is extremely sensitive to the size of what is assumed to be the true pool of ESEs (see below).

Analysing the full Ke-ESE dataset, rather than the reduced Ke-ESE400 dataset, we find that 18 out of 1,525 sequences (1.18%) are identified in all datasets. This is greater than expected, both as regards random sampling of all possible hexamers (expected number in common is 0 to 1, *P* <0.001) and from random sampling of the 1,525 nominated hexamers (expected number in common is 5 to 6, *P* <0.001). Curiously, it is also more than expected assuming that the 1,182 hexamers from Ke et al. are the complete population of true ESEs and the three smaller samples are random selections from this larger pool. In this instance we expect an intersection of only 11 to 12 hexamers, 18 being more than two standard deviations away. This latter result suggests that the three smaller candidate lists of ESEs are more similar than expected were the true list of ESEs the 1,182 discovered by Ke et al.. This suggests that either the Ke et al. longer list is identifying motifs that are not ESEs or that the methods are detecting different sorts of ESEs.

Assuming all methods are sampling from the same underlying pool of ESEs we can use this same approach to attempt to estimate the size of this pool of ‘true’ ESEs. To this end we varied the size of the pool that defined the complete list of ‘true’ ESEs. We then ask how big this pool size would have to be if, when we took four samples (of sizes 238, 238, 285 and 400), we found an intersection of size 10. The size of the ‘true’ ESE pool by this method is estimated to be around 850 hexamers.

The above estimate is strongly contingent on the assumption that all methods are sampling from the same underlying set of true ESE hexamers. If this is true, we expect when considering each pair of samples, that the datasets should be more similar than expected by chance. To this end we consider the overlap between each dataset and compare this against a null of independence, with *P* determined by simulation (Table 1). We see good concordance between some datasets. Notably, there is five-fold more overlap between RESCUE and PESE than expected by chance. Strikingly, ESR and Ke-ESE and Ke-ESE400 show no more overlap than expected by chance, the latter not being significant even before Bonferonni correction. Ke et al. report concordance between Ke-ESE and ESR.

**Table 1:** The extent of overlap between each dataset in pairwise combination

**Dataset 1**	**Dataset 2**	**n 1**	**n 2**	**O**	**E**	**F**	**Z**	***P *** **value**
RESCUE	PESE	238	238	75	13.8	5.42	17.3	**< 0.001**
RESCUE	ESR	238	285	55	16.6	3.32	10.1	**< 0.001**
RESCUE	Ke-ESE400	238	400	54	23.2	2.32	6.8	**< 0.001**
PESE	ESR	238	285	48	16.6	2.90	8.9	**< 0.001**
PESE	Ke-ESE400	238	400	65	23.2	2.80	9.2	**< 0.001**
ESR	Ke-ESE400	285	400	33	27.8	1.19	1.0	0.12195
RESCUE	Ke-ESE	238	1182	125	68.7	1.82	8.2	**<0.001**
PESE	Ke-ESE	238	1182	137	68.7	1.99	9.9	**<0.001**
ESR	Ke-ESE	285	1182	98	82.2	1.19	2.1	0.015

n1 = number of motifs in dataset 1; n2 = number of motifs in dataset 2; O = number of motifs in common between dataset 1 and dataset 2; E = expected = (n1 * n2)/T; where T is the total number of possible hexamers, that is, 4,096; F = overlap factor = O/E; factor >1 indicates more overlap than expected of two independent groups. Z score is the difference between O and E normalised by the standard deviation (derived from simulation). *P* values in bold are those significant after Bonferonni correction assuming P <0.05/9.

Given that each dataset is likely to include false positives, we considered two new intersect datasets, INT2 (316 motifs) and INT3 (84 motifs), composed of those motifs that appear in at least two or at least three sets respectively. Similarly, we also described INT2.400 (192 motifs) and INT3.400 (54 motifs) considering the Ke-ESE400 set. We assume that the trends in the overlap sets best reflect the properties of true ESEs. For the rest of the paper we hence consider the INT3 and INT3.400 overlap sets as our best measure of an ESE gold standard, in terms of their various properties and so compare all raw datasets against this gold standard. The intersection sets of hexamers are presented in Additional file 1: Table S1.

Both three-way intersection datasets are significantly larger than expected by a random null (*P* <0.001). To investigate the relative contribution of each raw dataset to the three-way intersection datasets we also consider a null simulation. We do not expect all raw datasets to be at equal proportions within the intersections datasets as the largest the intersection set can be is the size of the smallest group. Hence the largest raw dataset is likely to have the smallest proportional contribution to the intersection groups. To examine relative contributions then, we consider instead the extent of the deviation from null for each group. To this end we simulated four datasets in which we extracted, at random, the same number of hexamers as seen within each raw dataset. For each round of simulation we then determine how many hexamers from each group feature in the three-way (or greater) intersection group. Repeating this simulation 10,000 times we then determine the expected mean and standard deviation of the number of hexamers from each group in the intersection sets. We then employ a Z score to determine the deviation of the observed number within each set that is seen in INT3 and INT3.400. The Z-score is the difference between the observed and the expected number with the intersection dataset, normalised in standard deviation units. Results are shown in Additional file 2: Table S2. As expected all groups have very many more representatives in the intersection datasets than expected by chance. The deviation is highest for RESCUE and PESE and lowest for ESR. This may reflect ESR’s capture of some exonic splice inhibitors.

### Strong purine enrichment is seen in intersect datasets but not in all raw datasets

Many of the first identified mammalian ESE motifs were described as purine-rich motifs, although subsequent analyses suggest that this need not be true of all motifs. Likewise, SR proteins have been well documented as preferring purine-rich binding sites [2,24]. From this information we could expect the datasets to be enriched in adenine and guanine.

We computed the nucleotide content of all datasets (Table 2). Although each dataset shows dissimilar proportions of base usage, two of them (RESCUE and PESE) are, as expected, highly enriched for purines (Table 2). It is noteworthy that there is also strong purine enrichment (range, 63% to 82%) in all the four intersection groups. RESCUE has the closest resemblance to the three-way intersect groups.

**Table 2:** Nucleotide content of the ESE datasets

	**RESCUE**	**PESE**	**ESR**	**Ke-ESE400**	**Ke-ESE**	**INT2**	**INT3**	**INT2.400**	**INT3.400**
A	0.478	0.34	0.277	0.221	0.212	0.345	0.466	0.398	0.497
G	0.252	0.299	0.246	0.333	0.318	0.288	0.317	0.299	0.321
T	0.13	0.134	0.249	0.138	0.179	0.162	0.099	0.13	0.074
C	0.14	0.228	0.229	0.308	0.29	0.205	0.117	0.173	0.108
AG	0.73	0.639	0.522	0.554	0.530	0.633	0.784	0.697	0.818
GC	0.392	0.527	0.475	0.641	0.609	0.493	0.435	0.472	0.429
*P*	**4.1e-70**	**3.4e-26**	0.035	**6.1e-08**	**1.e-07**	**1.6e-31**	**2.2e-39**	**5.3e-42**	**1.3e-32**

*P* value is for a binomial test on purine content using absolute counts of nucleotides with a null of 50%. Data in bold is significant after Bonferonni correction (*P* <0.05/9).

There is some discrepancy between all intersect groups and the set of ESEs reported experimentally by Ke et al. which is far less enriched in adenine, having less than half the A content of the three-way intersect groups. The two Ke et al. datasets are only weakly enriched in purine (53% to 55%) and in this regard are comparable to ESR (52%).

An alternative question is how the purine content of the ESEs found at the ends of exons compares with the purine content of sequence at exonic ends not considered to be ESE. Here we find that all datasets support a strong difference between ESE and non-ESE (Table 3). Strikingly Ke-ESE400 in this analysis has a purine content over 60%.

**Table 3:** Purine content in and out of ESEs within the 50 bp at both exon ends

	**Mean:ESE**	**Mean:non-ESE**	**Median:ESE**	**Median:non-ESE**
ESR	0.5441842	0.5000044	0.5416667	0.5
INT2	0.6063711	0.4571333	0.6122449	0.4561404
INT2.400	0.6612339	0.4584617	0.6666667	0.4571429
INT3	0.7409292	0.4755146	0.75	0.4754098
INT3.400	0.7881002	0.4873524	0.8125	0.4871795
Ke-ESE	0.5532219	0.4672781	0.5529412	0.4655172
Ke-ESE400	0.6162153	0.4861662	0.6206897	0.484375
PESE	0.6167329	0.4687956	0.6190476	0.4693878
RESCUE	0.705773	0.4359836	0.7142857	0.4361702

We performed a paired Wilcoxon test for each exon comparing purine content in ESE and non-ESE. All tests are highly significant before and after Bonferonni correction and not shown.

### ESEs enrichment near exon ends is seen in the intersect datasets but not in all raw datasets

It is generally supposed, for good reason [9-11], that ESEs are located close to the intron-exon boundaries within exonic sequences and are avoided in intronic sequence. Consequently, we expect to find a higher frequency of motifs within exons than within their flanking introns and a higher frequency at the ends of the exonic sequences compared with the centre of the exons.

All datasets show a higher density of ESE hexamers in exons than introns, as expected (Table 4). Note that for many datasets this is circular as the ESEs were in part defined by such enrichment. Most datasets, including all intersect datasets, show that ESEs are enriched at exon ends compared to exon cores (Table 4). No datasets were defined by this property so this is not circular. Remarkably, Ke-ESE 400 and Ke-ESE both indicate that ESEs are more enriched at exonic cores, this result being significant even after Bonferonni correction.

**Table 4:** Distribution of ESEs between introns, exon core and exon flanks

**Dataset**	**Intron density median**	**Exon density median**	**Intron density median per hexamer**	**Exon density median per hexamer**	***P***^**a**^	**Ends density median**	**Core density median**	**Ends density median per hexamer**	**Core density median per hexamer**	***P***^**b**^
ESR	0.453	0.533	0.0016	0.0019	**2.4e-56**	0.541	0.524	0.0019	0.0018	**6.96e-11**
INT2	0.310	0.447	0.001	0.0014	**3.0e-60**	0.460	0.437	0.0015	0.0014	**1.8e-15**
INT2.400	0.201	0.305	0.001	0.0016	**3.8e-61**	0.327	0.298	0.0017	0.0016	**1.06e-16**
INT3	0.106	0.171	0.0016	0.002	**2.29e-61**	0.187	0.165	0.0023	0.0019	**1.59e-17**
INT3.400	0.069	0.117	0.0013	0.002	**2.7e-62**	0.131	0.113	0.0024	0.0021	**1.2e-18**
Ke-ESE	0.503	0.701	0.0004	0.0006	**2.1e-62**	0.697	0.706	0.0006	0.0006	**5.91e-20**
Ke-ESE400	0.161	0.308	0.0004	0.0008	**1.03e-64**	0.307	0.309	0.0007	0.0008	**0.0006**
PESE	0.256	0.383	0.0011	0.0016	**2.45e-59**	0.385	0.378	0.0016	0.0016	**0.0003**
RESCUE	0.217	0.286	0.0009	0.0012	**1.4e-60**	0.326	0.281	0.0014	0.0012	**7.0e-21**

Analysis of the ESE density in introns and in exon cores and flanks, where introns are defined by the terminal 100 bases (200 bp in total) and exons flanks are the 50 bp at either end and 100 bp in the centre of the exon.

^a^*P* value for Mann Whitney U test comparing ESEs between intron flanks and exon (50 bp first and last bases and 100 in the middle).

^b^*P* value for the Mann Whitney U test comparing exon flanks and exon cores. We also provide data on hexamer density per hexamer to enable visual comparison between different datasets but these were not employed for any statistics. *P* values in bold are significant after Bonferonni correction, with P <0.05/9.

This analysis has, however, a potential problem in that we are not directly comparing exon cores and exon ends of the same exons. If there are biases in composition of long exons, for example, this might bias results. To avoid this problem, we repeated the analysis using exons >200 bp with the central 100 bp being the core, the 50 bp at the flanks being considered in totality (*n* = 3,494 exons). We then performed a one-sided paired test to examine the hypothesis that ESEs are more common in the exon flank compared with the exon core of the same exon (Table 5). RESCUE-ESE once again strongly supports this hypothesis (Wilcoxon paired test, *P* = 8 × 10^-25^). The intersect datasets are the next most significant, followed by PESE all of which remain significant after Bonferonni correction. Ke-ESE shows no trend even before multitest correction (*P* = 0.46). A sign test largely supports the same conclusions. After Bonferonni correction, only RESCUE-ESE, PESE and the intersection datasets remain significant in both tests (Table 5).

**Table 5:** Paired test to examine the hypothesis that the ESE usage at exon flanks is higher than at the core of the same exon

**Dataset**	***P *- Wilcoxon paired test, one tailed**	***P *- sign test**
ESR	0.0067	0.016
INT2	**5.826948e-10**	**5.84E-07**
INT2-400	**2.275268e-14**	**3.41E-11**
INT3	**1.378492e-12**	**1.28E-09**
INT3-400	**8.045803e-12**	**5.82E-10**
Ke-ESE	0.46	0.81
Ke-ESE400	**0.00079**	0.013
PESE	**0.00012**	**0.00358**
RESCUE	**7.755973e-25**	**1.80E-15**

Here we compare the first and last 50 bp of an exon with 100 bp in the centre of the exon (using exons >200 bp, *n* = 3,494). Only those in bold are significant after Bonferonni correction (with P <0.05/9).

### RESCUE and intersect datasets predict differences in synonymous codon usage

The above tests consider the absolute usage of a codon in various compartments (exon core, intron, exon flank). We also expect that, as ESEs are expected to be employed more towards exon ends, their usage should increase close to exon ends. This can be assayed by considering the slope on the regression line relating proportional codon usage to distance from a boundary, a negative slope implying increased usage near exon ends. Indeed, as one approaches exon boundaries the usage of GAA relative to its synonym GAG increases [26]. This was suggested to reflect the greater usage of GAA compared to GAG in ESEs coupled with the increasing usage of ESEs at exonic ends. A more systematic analysis of the RESCUE-ESE set of hexamers revealed that this trend is generally true in a great excess of instances, that is, a codon that is more commonly found in ESEs (defined by RESCUE-ESE) is increasingly preferred over its synonym as one moves closer to exon junctions [27]. Do the other datasets provide similar results and are the intersection datasets comparable?

To address this, as before, we define for each codon a Z score, the hexamer preference index, reflecting the extent to which the codon is found in any given set of ESEs compared with expectations derived from the usage of that codon in the genome (Additional file 3: Table S3). Via this metric we can both ask which of two synonymous codons are more commensurate with ESE function, given any particular ESE set, and which are relatively preferred near boundaries. We consider all 87 possible pairwise comparisons between synonymous codons (for example, we compare GAA to GAG in both HPI and slope of usage as one approaches boundaries).

To this end we derived, for each codon, the slope of the proportional usage of that codon as a function of the distance from the exon boundary. We then consider the difference in slope between two synonymous codons and the difference in HPI between the same two. Importantly, we orient all calculations such that the difference in HPI is always positive. Owing to this orientation, there are two expectations. First, we expect for real ESEs that the majority will have a negative difference in slope, that is, the codon more preferred in ESEs should also be the codon more preferred (or less avoided) near exon boundaries. For example, if GAA is more common in ESEs than GAG (HPI for GAG is greater than that for GAG), then the slope on the line of GAA usage as a function of the distance from the exon boundary should be strongly negative, while that for GAG should be less negative, or potentially positive. If GAA is more preferred in ESEs and thus has a higher HPI score, we thus consider:

Differenceinslope=slopeforGAA–slopeforGAG.

If then HPI predicts the slopes this difference should be negative. This we examine via a binomial test with a 50:50 null.

Second we expect there to be a negative correlation between the difference in HPI and the difference in slope: if two codons are greatly discordant in HPI, there should also be a great discordance in slope, with the one more preferred in ESEs more preferred near boundaries. Were GAA, for example, strongly enriched in ESEs it should also be strongly preferred near boundaries. It should have a high HPI and strongly negative slope. If GAG is less enriched in ESE it should have a lower HPI and a lower slope. If the difference in HPI is marked, the difference in slope should also be marked. Hence we expect a negative correlation between the difference in slope (which should be strongly negative given the orientation of the difference) and the difference in HPI, which is always positive.

In principle a dataset could fail test 1 and pass test 2 or *vice versa*. For example, it may be the case that all codons preferred in ESE are avoided near boundaries (fail test 1), while at the same time, within the data there is an overall negative trend. Thus these can be considered as two independent tests. We combine the *P* values derived from these two tests using Fisher’s method to derive one overall *P* value.

**Figure 2:**
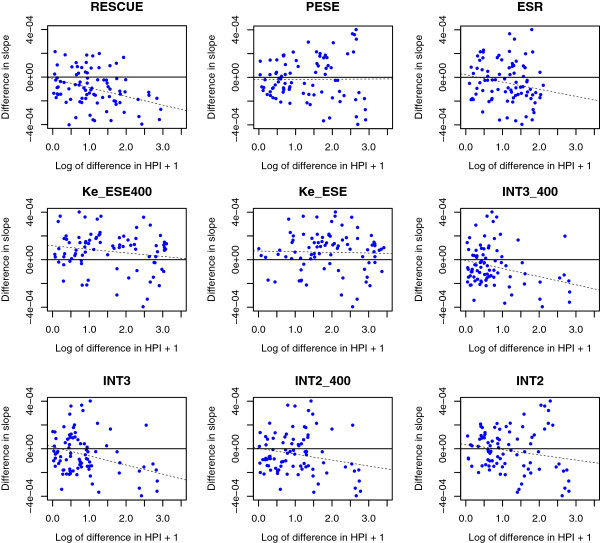
**The ability of each set of ESEs to predict trends in relative synonymous codon usage.** We plot the difference in HPI *versus* the difference in slope of codon usage, as one approaches a boundary for all pairs of synonymous codons. A negative slope implies a codon is enriched near boundaries. Thus we expect those codons with a high HPI to have a more negative slope. Thus we expect a large difference in HPI to be reflected in a large negative difference in slope between two synonymous codons.

Once again, RESCUE ESE is consistent with both predictions and the overall significance is very high (P <10^-7^) (Figure 2; Table 6). The intersect datasets are likewise significant, although INT2 is not robust to Bonferonni correction, but all others are. And once again, however, Ke-ESE and Ke-ESE400 go against expectations. Both suggest that codons relatively preferred near boundaries are less preferred in ESEs. This in part accords with the unexpected result that their ESEs are enriched in exon cores. ESR is overall significant while PESE is not, although the binomial test goes in the expected direction.

**Table 6:** The ability of each set of ESEs to predict trends in relative synonymous codon usage

**Dataset**	**Negatives**	**Positives**	***P *** **binomial**	**Rho**	***P *** **corr**	**Chi**^**2**^	***P *** **overall**
ESR	54	33	0.016	-0.21	0.056	14.04	**<0.001**
INT2	46	41	0.33	-0.18	0.1	6.82	<0.05
INT2.400	57	30	0.0025	-0.14	0.2	15.2	**<0.0005**
INT3	56	31	0.0048	-0.24	0.027	17.90	**<0.0005**
INT3.400	60	27	0.00026	-0.18	0.1	21.11	**<0.0001**
Ke-ESE	23	64	1	0.0015	0.99	0.02	ns
Ke-ESE400	23	64	1	-0.091	0.4	1.83	ns
PESE	49	38	0.14	0.00033	1	3.93	ns
RESCUE	66	21	7.10E-07	-0.31	0.0031	39.9	**<0.0000001**

Here was ask whether: (a) each ESE dataset can predict which of two synonymous codons is preferred near a boundary and which is relatively preferred in ESEs, assayed by their HPI scores; and (b) whether the extent of the difference in tendency to be found in ESEs predicts the degree of difference in the preference as one approaches exon ends. Regarding the first aspect, the expectation is that, orientating all comparisons such that the difference in HPI >0, the difference in slope should be negative. We thus ask whether there are more negative values than positives under a directional binomial test. As regards issue (2), we expect a negative correlation: a codon strongly preferred in ESE should be relatively strongly enriched near a boundary, hence a big difference in the slope of the codon usage near the boundary. We compute an overall *P* value combining the *P* values of the two tests using Fisher’s method to generate a chi squared value, with 2 degrees of freedom. Those indicated in bold are significant after Bonferonni correction (P <0.05/9).

### All datasets, except those of Ke et al., support slower evolution of ESEs

It is to be expected that synonymous mutations within functioning ESEs are subject to purifying selection. Since conserving the ESE motifs is important for proper splicing, we expect a higher number of synonymous substitutions in non-ESE sequences than in sequences involved in ESE activity. This has been confirmed previously for RESCUE [6] and is definitional for ESR.

To check this behaviour, each exon was split in two and the human-mouse alignments were computed (see methods). Every human nucleotide within the alignment was designated as ESE or non-ESE according to a given dataset. We consider every codon in frame moving towards the interior of an exon. We consider the fate of four-fold degenerate sites exclusively (that is, third sites in codons in which the codon is a member of the same four-fold degenerate codon group in both species). We first considered those sites that we considered to be within an ESE and calculated the proportion of such sites that have changed between the two species. We then considered the same for sites considered not to be ESE.

**Figure 3:**
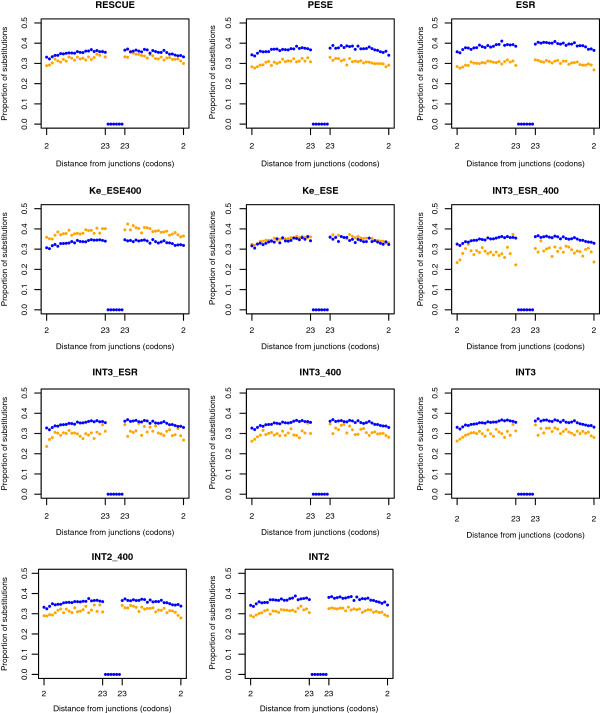
Rate of evolution of ESE and non-ESE sequence as a function of the distance from an exon boundary.

As ESR is defined by the need for the motifs to be slowly evolving at third sites, analysis for this set is circular (for visual purposes alone we present the result in Figure 3). More importantly, for the intersect datasets we generate two new intersect sets, these being the three-way intersection between RESCUE, PESE and Ke-ESE, which we term INT3_ESR (*n* = 51) and between RESCUE, PESE and Ke-ESE400, which we term INT3_ESR_400 (*n* = 30) (Additional file 1: Table S1 for lists of hexamers). This avoids the circularity that the hexamers must in part be slow evolving owing to the inclusion of the ESR hexamers.

As can be seen in nearly all datasets (Table 7a; Figure 3), ESEs evolve slower than non-ESE sequence that is equidistant from an exon-intron junction. We test the difference by considering a paired (by distance to the junction) Wilcoxon test (Table 7a). ESEs in all samples evolve slower, including the two new intercept datasets, except for those specified by Ke-ESE and Ke-ESE400 which are, once again, the deviant set in having significantly higher, not lower, rates of evolution. This result contradicts the claim of conservation of motifs made by Ke et al.. They, however, asked about the presence of the hexamer in macaques rather than the rate of substitution within the motifs.

**Table 7:** Rate of evolution of ESE and (a) non-ESE sequence and (b) pseudoESE sequence at four-fold degenerate sites

**Dataset**	**Median ESE**	**Median non-ESE**	**% Difference**	***P *** **two-sided**
A				
ESR	0.3	0.39	-23.1	**1.10E-13**
INT2.400	0.32	0.36	-11.1	**1.10E-13**
INT3	0.3	0.36	-16.7	**1.10E-13**
INT3.400	0.3	0.35	-14.2	**1.10E-13**
INT3_ESR	0.3	0.35	-14.2	**1.10E-13**
INT3_ESR_400	0.29	0.35	-17.1	**3.40E-13**
Ke-ESE	0.35	0.34	2.9	**8.20E-08**
Ke-ESE400	0.38	0.34	11.8	**1.10E-13**
PESE	0.31	0.37	-16	**1.10E-13**
RESCUE	0.32	0.35	-8.5	**5.70E-13**
B				
ESR	0.3	0.35	-14.3	**4.50E-13**
INT2.400	0.32	0.35	-8.5	**4.50E-13**
INT3	0.3	0.35	-14.3	**4.50E-13**
INT3.400	0.3	0.34	-11.8	**4.50E-13**
INT3_ESR	0.3	0.34	-11.8	**4.50E-13**
INT3_ESR_400	0.29	0.34	-14.7	**2.30E-12**
Ke-ESE	0.35	0.35	0	**4.10E-06**
Ke-ESE400	0.38	0.36	5.6	**4.50E-13**
PESE	0.31	0.35	-11.4	**4.50E-13**
RESCUE	0.32	0.35	-8.5	**4.50E-13**

We consider the proportion of four fold degenerate sites that are changed or unchanged when comparing mouse-human alignments. We split sites by whether in human the sequence matches an ESE or not (Table A) or ESE *versus* pseudoESE (Table B). We then perform a Wilcoxon paired test considering the rate of evolution of ESE and non-ESE or pseudoESE at each position away from an exon boundary. We also present the % difference between the medians, this being (median for ESE - median for non-ESE)/median for non-ESE. All tests are significant after Bonferonni correction (for consistency the *P* values are shown in bold).

The above analysis is, however, potentially confounded by differences in nucleotide content between ESE and non-ESE sequence. To address this, for all ESE sets we considered the nucleotide content of the ESEs and generated random hexamers with, on average, the same nucleotide content. We generated as many random hexamers as there are hexamers in each set. We then perform the same test as above but replacing the real hexamers with the pseudoESE set. For each ESE set we repeated this process 100 times (generating a diversity of pseudoESEs in each case) and considered the median value at each distance from a boundary. We then repeated the paired Wilcoxon test as above comparing the rate of evolution for the real hexamers with that of the nucleotide matched pseudohexamers. Any differences cannot be owing to nucleotide content differences as this is controlled. The rate of evolution of the pseudohexamers is consistently higher than that of the real hexamers, excepting for the case of the two Ke at al. datasets (Table 7b). These results thus support the conclusions of the original nucleotide-uncontrolled analysis. They indicate that the lower rate of evolution of ESEs cannot be accounted for in terms of skewed nucleotide content and thus most likely reflect differences in the strength of purifying selection.

### All datasets, except those of Ke et al., support SNP rarity in ESEs

**Figure 4:**
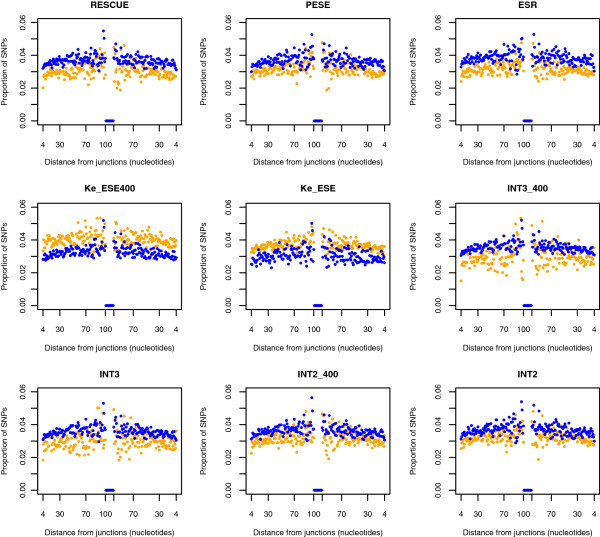
**SNP density in and out of ESEs as a function of the distance from the exon boundanry.** ESE data is in orange, non-ESE data in blue.

To further scrutinise these results and to check that they are not an artifact of mouse-human analysis, we asked whether ESEs are less likely to harbour SNPs within the human population [7,11]. As above we find that all datasets, bar the two derived by Ke et al., strongly support a rarity of SNPs in ESEs as opposed to non-ESE that is equidistant from exon junctions (Table 8a; Figure 4). We recapitulate the finding that SNPs are rare towards exonic ends [11]. This is consistent with purifying selection acting stronger in ESEs than in non-ESE.

**Table 8:** The proportion of sites with a SNP depending on distance from an exon boundary (a) comparing ESE and non-ESE and (b) comparing ESE and nucleotide matched pseudoESE

**Dataset**	**Median ESE**	**Median non-ESE**	**% Difference**	***P *** **two-sided**
A				
RESCUE	0.03	0.036	-16.7	**1.60E-28**
PESE	0.031	0.036	-13.9	**7.10E-27**
ESR	0.031	0.038	-18.4	**8.30E-30**
Ke-ESE400	0.04	0.032	25	**6.20E-30**
Ke-ESE	0.036	0.03	20	**1.20E-31**
INT3.400	0.029	0.035	-17.1	**1.30E-22**
INT3	0.029	0.035	-17.1	**9.60E-25**
INT2.400	0.031	0.036	-13.9	**2.10E-24**
INT2	0.032	0.036	-11.1	**4.10E-23**
B				
RESCUE	0.03	0.031	-3.2	**2.70E-18**
PESE	0.031	0.033	-6.1	**7.80E-18**
ESR	0.031	0.034	-8.8	**2.30E-26**
Ke-ESE400	0.04	0.036	11.1	**1.60E-26**
Ke-ESE	0.036	0.035	2.9	**7.30E-18**
INT3.400	0.029	0.034	-14.7	**4.20E-24**
INT3	0.029	0.035	-17.1	**5.70E-27**
INT2.400	0.031	0.035	-11.4	**1.20E-27**
INT2	0.032	0.035	-8.6	**1.80E-28**

Statistics are from a paired test in which we compare the proportion of sites with a SNP within an ESE with sites equidistant from an exon junction but (a) not in an ESE or (b) in a pseudoESE, using a Wilcoxson paired test. Percentage difference is defined as (Median for ESE -Median for non-ESE)/Median for non-ESE. All tests are significant after Bonferonni correction (for consistency the *P* values are shown in bold).

To be confident that the above result is not an artifact of differential nucleotide content, we performed a nucleotide-controlled analysis, comparable to the above analysis. We generated pseudo hexamer sets with the same average nucleotide content as each focal ESE set and the same number of hexamers as the focal set. We then matched these to the exons and asked, for each distance from a boundary, about the proportion of pseudo ESE sites with SNPs and compared this to the real hexamers. All results are unaffected (Table 8b) with Ke-ESE datasets having higher SNP rates than the nucleotide control set and all others having lower SNP rates in ESEs than in pseudoESEs. The magnitude of the difference between ESEs and the pseudoESEs is typically lower than that between ESEs and non-ESEs. This might be owing to differences in mutability between pseudoESE and non-ESE, owing to nucleotide effects, or because the pseudo sets contain numerous true ESEs.

### All datasets support high ESE density in the vicinity of weaker splice sites

The RESCUE-ESE dataset assumed that ESEs would be enriched in proximity to weaker splice sites. Do other datasets support such a compensatory relationship, wherein weak splice sites are buffered by a greater density of ESEs? To this end we calculated splice site strength (using the maximum entropy model of MaxEntScan [40]) and considered the correlation between this and the density of ESEs within the terminal 50 bp (omitting the terminal 3 bp from exon ends). We considered 5’ and 3’ ends separately and also the merged set. To consider the intersection datasets, as above, given the circularity of employing RESCUE-ESE we define new interaction datasets INT3_RESCUE and INT3_RESCUE_400 that omit the RESCUE ESE data. All datasets concur that weak splice sites are matched by a higher density of ESEs in the flanking exon (Table 9). While this is a definitional feature for RESCUE-ESE, it might also be definitional for the Ke et al. datasets as the exon ends of this experimental study were modified to reduce their accuracy, thereby permitting assessment of the compensatory effect of all hexamers.

**Table 9:** The correlation between splice site strength and ESE density at 5’ and 3’ ends of exons

	**5’**		**3’**	
**Dataset**	**rho**	***P***	**rho**	***P***
ESR	-0.047	**5.06E-19**	-0.059	**3.90E-29**
INT2	-0.098	**1.50E-77**	-0.108	**8.56E-93**
INT2.400	-0.099	**6.63E-78**	-0.092	**2.21E-68**
INT3	-0.093	**9.12E-69**	-0.075	**3.38E-46**
INT3.400	-0.094	**6.76E-71**	-0.070	**9.01E-40**
INT3_ESR	-0.091	**1.25E-66**	-0.060	**8.53E-30**
INT3_ESR_400	-0.083	**2.61E-56**	-0.054	**3.43E-24**
INT3_RESCUE	-0.078	**2.11E-49**	-0.079	**1.98E-50**
INT3_RESCUE_400	-0.074	**1.64E-44**	-0.065	**1.93E-34**
Ke-ESE	-0.075	**3.73E-46**	-0.129	**8.84E-132**
Ke-ESE400	-0.080	**9.16E-52**	-0.126	**3.95E-126**
PESE	-0.082	**2.26E-54**	-0.097	**7.87E-75**
RESCUE	-0.099	**5.41E-79**	-0.054	**1.66E-24**

All *P* values are significant after Bonferonni correction (shown in bold for consistency).

### Most datasets find no difference in ESE density between alternative and constitutive exons and between conserved and non-conserved exons

It has been reported that alternative exons, if they have an orthologous exon in other species, tend to be slow evolving [6,30,31]. We find the same. Alternative exons with a mouse ortholog evolve slower at synonymous sites at both ends (5’ end *P* = 0.0026, 3’ end *P* = 0.0006) and at non-synonymous sites (5’, *P* = 5 × 10^-8^, 3’ *P* = 4 × 10^-5^) than constitutive exons. Might this reflect a higher density of ESEs in alternative exons? Do conserved exons have a higher density than non-conserved ones?

Considering alternative *versus* constitutive exons (as defined by MGI), there is general agreement that the two classes do not differ in ESE density in the final 100 bp (Table 10). However, we note one peculiarity, namely that the Ke et al. datasets find strong evidence for ESE density being lower in alternative exons, as previously noted [20]. This is significant for Ke-ESE after Bonferonni correction. PESE concurs with this result at 5’ but not 3’ ends. Over-representation in constitutive exons was, however, definitional so this is likely a method artifact. RESCUE-ESE finds a weak trend in the reverse direction not significant after multi-test control. This may also be a method artifact [18]. A lower density of ESE motifs in alternative exons cannot explain the slower evolution of exon ends in alternative exons, unless ESE motifs are fast evolving, which appears not to be the case.

**Table 10:** ESE density in alternative and constitutive exons

	**Two-tailed**		**Alternative > constitutive**		**Alternative < constitutive**	
**Dataset**	**end5**	**end3**	**end5**	**end3**	**end5**	**end3**
ESR	0.84	0.15	0.42	0.92	0.58	0.08
INT2	0.059	0.21	0.97	0.90	0.03	0.10
INT2.400	0.48	0.34	0.76	0.83	0.24	0.17
INT3	0.325	0.832	0.84	0.59	0.16	0.41
INT3.400	0.989	0.8361	0.49	0.58	0.51	0.41
Ke-ESE	**2.46E-006**	**0.00065**	0.99	0.99	**1.23E-006**	**0.00032**
Ke-ESE400	0.027	0.019	0.99	0.99	0.014	0.0096
PESE	**0.0012**	0.022	0.99	0.99	**0.00061**	0.0111
RESCUE	0.029	0.0413	0.015	0.02	0.99	0.98

Numbers represent *P* values from Mann Whitney U test comparing ESE density in alternative and constitutive exons. We present the results of the two-tailed test and the two alternative one-tailed tests. *P* values in bold are significant after Bonferonni correction (P <0.05/18). We compare 8,406 alternative exons and 3,249 constitutive ones.

As regards conserved versus non-conserved exons, nearly all datasets suggest no difference (Table 11). However Ke-ESE hexamers are more enriched at 3’ ends of conserved exons than in non-conserved exons. Ke-ESE400 does not support this conclusion.

**Table 11:** ESE density in conserved and non-conserved exons

	**Two-tailed**		**Conserved > non**		**Conserved < non**	
**Dataset**	**End 5’**	**End 3’**	**End 5’**	**End 3’**	**End 5’**	**End 3’**
ESR	0.91	0.78	0.45	0.39	0.55	0.61
INT2	0.36	0.15	0.82	0.075	0.18	0.92
INT2.400	0.18	0.41	0.91	0.21	0.09	0.79
INT3	0.98	0.67	0.49	0.66	0.51	0.34
INT3.400	0.53	0.61	0.74	0.69	0.261	0.31
Ke-ESE	0.09	0.0029	0.044	**0.00147**	0.96	0.99
Ke-ESE400	0.86	0.34	0.43	0.17	0.57	0.83
PESE	0.43	0.45	0.78	0.22	0.21	0.78
RESCUE	0.23	0.047	0.88	0.98	0.12	0.024

Numbers represent *P* values from Mann Whitney U test comparing ESE density in conserved and non-conserved exons. We present the results of the two-tailed test and the two alternative one-tailed tests. *P* values in bold are significant after Bonferonni correction (*P* <0.05/18). We compare 3,413 conserved exons and 3,249 non-conserved ones. Conservation is defined by presence/absence in mouse.

### Most datasets agree that ESE density is highest when the neighbouring intron is large

Just as exons with weak splice sites are those requiring more ESEs, probably because these need more aid in specifying splice location, so too we might expect that exon ends flanked by larger introns should be harder to accurately identify. In support of this, it has been observed that for exons flanked by introns up to about 1.5 kb in length there is a correlation between intron length and ESE density using RESCUE ESE motifs [29]. We replicate this finding and find that most intersect datasets confirm it (Table 12). The same trends are seen when considering all exons, no matter what the size of the flanking intron (Table 12). Once again, in both incidences, the Ke et al. datasets indicate the opposite trends.

**Table 12:** The correlation between ESE density and the size of the flanking intron, for exons with flanking introns <1,501 bp (columns 1 and 2), and for all exons (columns 3 and 4)

	**Introns < 1500 bp**		**All**	
**Dataset**	**r**	***P***	**r**	***P***
ESR	0.04459	**3.02e-06**	0.026	**8.44E-05**
INT2	0.01595	9.5e-02	0.012	0.0712
INT2.400	0.04758	**6.24e-07**	0.048	**1.31E-13**
INT3	0.05225	**4.41e-08**	0.051	**4.37E-15**
INT3.400	0.05107	**8.84e-08**	0.052	**2.61E-15**
Ke-ESE	-0.07193	**4.73e-14**	-0.083	**6.51E-37**
Ke-ESE400	-0.05551	**6.05e-09**	-0.052	**1.86E-15**
PESE	-0.00535	5.75e-01	-0.028	**1.96E-05**
RESCUE	0.10278	**0.00e + 00**	0.114	**2.97E-68**

*P* values in bold are significant after Bonferonni correction (*P* <0.05/18).

Are the trends as regards splice site strength and size of the neighbouring intron independent? To address this we consider ESE density predicted in a partial spearman correlation (Additional file 4: Table S4a; for raw correlations see Additional file 5: Table S5). As intron size and number vary as a function of expression parameters, with highly/broadly expressed genes tending to have numerous small exons and small introns, it is worthwhile adding expression parameters and exon size to the consideration (Additional file 4: Table S4a). We find that exons flanked by larger introns tend to have stronger splice sites. Allowing for this covariance we still report a higher ESE density for exons with weak splice sites. The relationship between intron size and ESE density appears to be robust to covariate control with larger introns being associated with a higher density of ESEs in all intersect datasets (although not significant after Bonferonni correct for INT2) and profoundly so in RESCUE-ESE. The two Ke et al. datasets in the covariate-controlled analysis again find the opposite trend. These results are largely unaffected by considering only those exons flanked by introns less than 1,500 bp Additional file 6: Table S4b). Expression parameters tend not to be important predictors of ESE density, although Ke-ESE suggests a higher density for highly expressed tissue specific genes. In the intersect datasets exon length is not a significant predictor.

## Discussion

Early detailed studies of binding sites for SR proteins and exonic splice enhancer elements suggested a view of ESEs that has remained largely consistent with most systematic analyses and analyses of where within exons mutations that affect splicing are found [10]. That is to say ESEs tend to be purine rich [9,21-23], enriched near exon ends [8-10] and slow evolving [5-7,10,11]. A feature of the enrichment near exon ends is that knowledge of ESEs enables one to predict relative synonymous codon usage [27]. In addition ESE density is higher near weak splice sites and near longer introns. Importantly, all of these conclusions are robustly supported by what one might consider to be the datasets with the lowest false positive rate, namely the two three-way intersection datasets.

### Estimating the impact of ESEs on the rate of evolution at synonymous sites

The intersection datasets suggest that ESEs have a synonymous rate of evolution around 15% lower than non-ESE sequence. The net impact of this depends on the proportion of sequence that is ESE and the proportion of sequence that is near exon ends. The mean proportion of sequence near exon ends that is ESE varies as a function of the total number of ESEs and, naturally, this is small for the intersect datasets. Of the non-intersect sets the density is approximately 30% (it is 25% including all datasets). This suggests that ESEs reduce synonymous substitution rate around 5% on average close to exon ends. With approximately 80% of sequence near exon ends, this means that the net impact of ESEs on synonymous rates of evolution is of the order of a 4% reduction. This is likely to be a conservative estimate as it is possible that sequence considered to be non-ESE is misclassified either because it really is non-ESE in human but is ESE in the comparator species, or because it has been incorrectly classified as non-ESE, this being an especially acute problem for the intersect datasets. If some sequence is classified as ESE but is really non-ESE, then this should also lessen the difference between non-ESE and true ESE. Our estimates of a 4% reduction is lower than recent estimates that circa 20% and 10% of synonymous sites are under selection in humans and mice, respectively [41]. As non-synonymous mutations can also be deleterious owing to modulation of splicing, we also estimate that at least 4% of non-synonymous mutations are deleterious owing to their impact on ESEs.

Given that slow evolution is a property of ESEs, should slow evolution be used as part of the definition when searching for ESEs, as done with ESR? While it is no surprise to find that indeed ESR hexamers are slow evolving, it is striking to note that they are no more slow evolving that several of the intercept datasets. Indeed, the slowest evolving set of hexamers is INT3_ESR_400, the intercept dataset that excludes ESR. Slow evolution of ESEs thus seems a robust conclusion. However, we caution against the methodology of ESR because, in part, the slow evolution of ESR can be attributed to the unusual nucleotide content of this set of hexamers, the nucleotide composition controlled set evolving much slower than the non-ESE sequence, this feature being peculiar to ESR. This suggests that simply identifying slow evolving sequence is not a robust method to identify ESEs, as it must enrich for motifs that are intrinsically slow evolving owing to a low mutation rate (owing to their nucleotide content), as well as enriching for those under purifying selection.

### Why is the dataset of Ke et al. different?

Of the original four datasets RESCUE-ESE is highly consistent both with prior expectations and with the behavior of the intersection datasets. What was not expected was that the first high-throughput fully experimental dataset [20] is not simply discordant, but behaves opposite to the consensus and to prior expectation: the hexamers are not strongly purine enriched, are enriched at exon cores over flanks, get the prediction of trends in codon usage reversed from what is seen, are faster evolving and contain a higher density of SNPs than non-ESE sequences and are not enriched near longer introns. Moreover the Ke et al. datasets suggest that ESEs are enriched in conserved constitutive exons a largely unreplicated result. *A priori* these are surprising results, as we would have expected that the first systematic fully experimental approach would have produced a gold standard dataset.

An open question is why these two datasets (Ke-ESE and Ke-ESE400) are so discordant. One possibility is that Ke et al. identify a set of motifs that modify splicing by mechanisms not revealed by the alternative analyses. Perhaps they have discovered motifs that are key to splice modification in exonic cores? Perhaps some of these motifs have more direct effects on splicing owing to some of them being so close splice sites? This possibility, considered by Ke et al., is bolstered by their analysis showing that the hexamers close to splice sites impact strongly on RNA structure (known to be important in splice site selection), and nucleosome occupancy. If indeed, the method of Ke et al. has enriched for motifs that function by mechanisms other than SR protein binding, owing to their proximity to splice sites, then their peculiarity might in part be expected. This possibility is bolstered by the finding that the ESEs identified by Ke et al. in their strongest set have a relatively low purine content (Table 2), while the same ESEs when identified within the terminal 50 bp of exons have a much higher purine content (over 60%: Table 3) compared to flanking sequence. This suggests that the Ke et al. method has identified some classical ESE motifs that function close to exon boundaries, but has, in addition, identified less purine rich motifs that are modulating splicing possibly by alternative mechanisms in other parts of exons.

We notice a further unusual property, potentially consistent with the above explanation for the oddity of the Ke et al. data. If we consider the nucleotide usage at each codon position for each set of hexamers, then we can define the information content at each site (Additional file 7: Figure S1). Notably, the two Ke et al. datasets are the only two in which information content increases monotonically across hexamers, that is, information content at base *i,* is always greater than information at base *i -*1 (Additional file 7: Figure S1). This might suggest that close proximity of the hexamer insertion sites (in one instance within 5 nucleotides of the 5’ splice site) has impacted on the sorts of hexamers that were recovered that promoted splicing.

The possibility that the motifs described by Ke et al. might be specialist to splice control at particular locations within exons could potentially explain some of the discrepancies that we see. Might it be, for example, that they have enriched for specialist motifs that promote splicing in an exon core, but that hinder splicing when closer to exon ends? Such motifs may be under selection to not be present at exonic ends. This might, in turn, explain the increased rate of evolution of the motifs close to exon ends and their enrichment in exon cores. The fast evolution would be positive selection to lose the inappropriately located splice modifier.

Even if the above rationalisation is true, it does not obviate the finding that most splice-associated mutations are at exon flanks [10], while the Ke et al. sets of motifs are enriched in exon cores. This indicates that the motifs described by Ke et al. are not an unbiased sample of the pool of splice-associated motifs. This is reason enough to caution against use of this dataset as a sole (unbiased) guide to the properties of ESEs on a genome-wide scale.

This still leaves the problem of how the bias might have come about. As all of the hexamers in the Ke et al. data are experimentally confirmed, they cannot be dismissed simply as false positives. The profile of which motifs one discovers might, however, be dependent on both the focus of interest of the researchers and the nature of the mini-genes employed. As regards the former possibility, Ke et al. unusually pay explicit attention to motifs that function more centrally in exons. This focus of interest might well have led to their reporting a disproportionate number of specialist exon core motifs, assuming such things exist. Given the current popularity of the mini-gene approach, the second possibility, that the design of a mini-gene impacts on the profile of motifs that are discoverable, is, we suggest, worthy of deep scrutiny. It is the nature of the mini-gene approach that, by definition, the constructs are relatively compact. Might this lead to inevitable biases? Consistent with this possibility we notice that the Ke at al. hexamers are unusual in being enriched in constitutive exons and rare in proximity to larger introns. With the possibility that the process of splicing for short and long introns is qualitatively different, the impact of the choice of mini-gene structure requires close experimental scrutiny. We suggest that a repeat of the analysis of Ke et al., but varying intronic dimensions, would be informative as to whether the set of motifs recovered is modulated by even simple properties of the mini-gene employed.

## Conclusions

The consensus ESE sets support the notion that ESEs are purine rich, most common at exonic ends (and capable of explaining skews in codon usage towards exon ends), subject to purifying selection and more common when the flanking intron is long and when the splice site is weak. Of the original datasets RESCUE-ESE captures these trends, while the recent experimental dataset of Ke et al. behaves in many regards opposite to the consensus. We estimate that approximately 4% of synonymous mutations are deleterious owing to alteration of splicing mediated by disruption of ESEs.

## Competing interests

The authors declare that they have no competing interests.

## Authors’ contributions

LDH and EFC devised the plan of research. LDH wrote the manuscript. EFC and LDH performed the analysis. All authors read and approved the final manuscript.

## Supplementary Material

Additional file 1: Table S1 The hexamers that feature in the various intersection datasets. Click here for file

Additional file 2: Table S2 Deviation of each raw dataset in the intersection datasets from null expectation measured as a Z score. Click here for file

Additional file 3: Table S3 HPI scores for all codons given each ESE dataset. Click here for file

Additional file 4: Table S4a Partial spearman correlations for the instances where exons are flanked by introns of any size. Click here for file

Additional file 5: Table S5 Raw correlations between ESE density and seven possible predictive variables. Click here for file

Additional file 6: Table S4b Partial spearman correlations for the instances where exons are flanked by introns smaller than 1,500 bp. Click here for file

Additional file 7: Figure S1 Information content across the hexamers in each dataset. Click here for file
