# Supplementary figures and images for: The evolution, impact and properties of exonic splice enhancers
Source: Genome Biol. 2013 Dec 20;14(12):R143. doi: 10.1186/gb-2013-14-12-r143 (PMC4054783; doi:10.1186/gb-2013-14-12-r143)

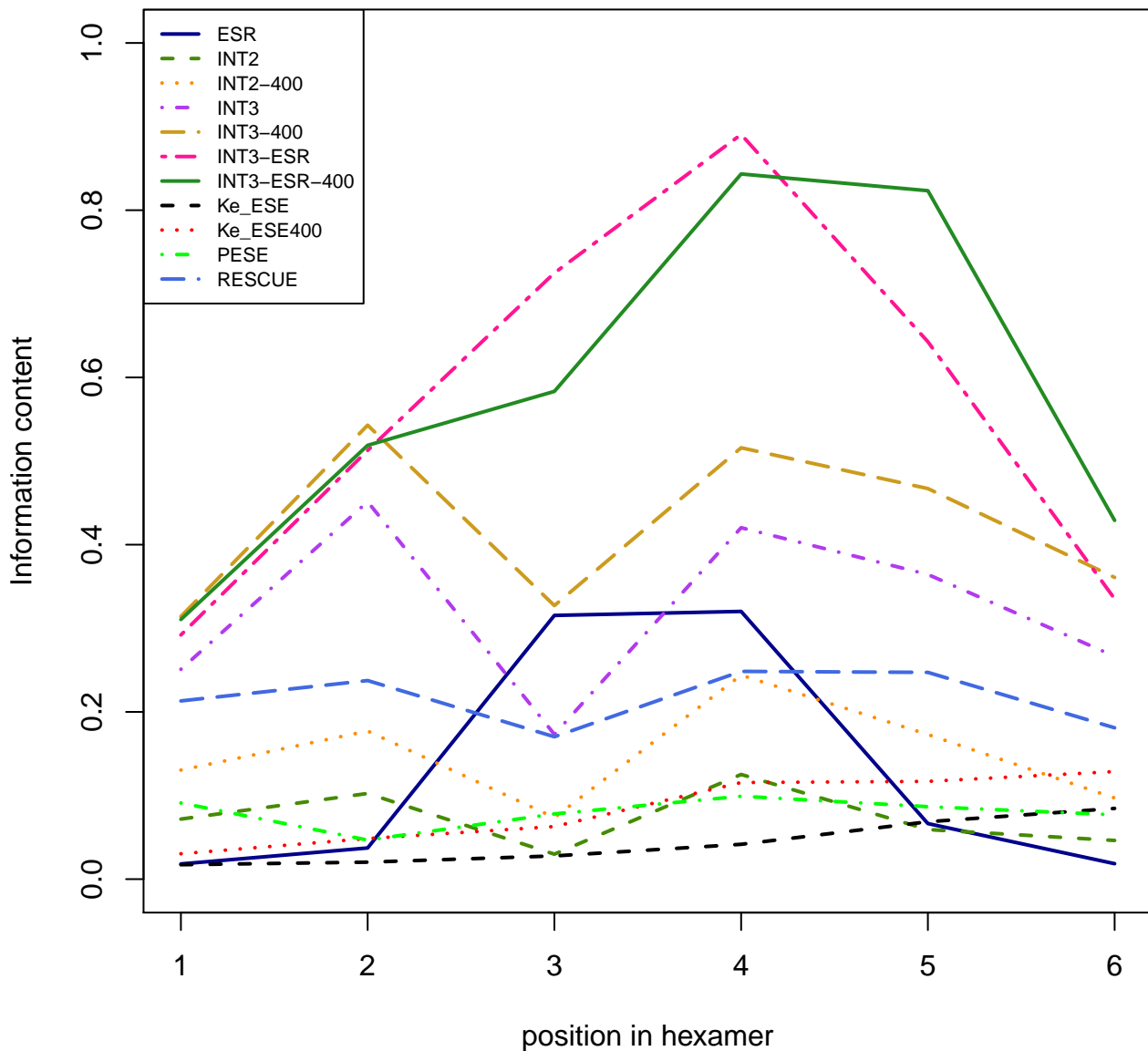

Supplement: Additional file 7: Figure S1 — Information content across the hexamers in each dataset. [file gb-2013-14-12-r143-S7.pdf]
